# Outbreak: Foodborne Illness and the Struggle for Food Safety

**DOI:** 10.3201/eid2512.191192

**Published:** 2019-12

**Authors:** Robert Tauxe

**Affiliations:** Centers for Disease Control and Prevention, Atlanta, Georgia, USA

**Keywords:** food safety, regulations, outbreak impact, change agents

Public health advances step by step, as hazards are recognized and better control and prevention strategies are developed. How this happens, how new safety measures come into being, and how they are improved and become part of the way we live are the focus of this new book, Outbreak: Foodborne Illness and the Struggle for Food Safety (Figure).

**Figure:**
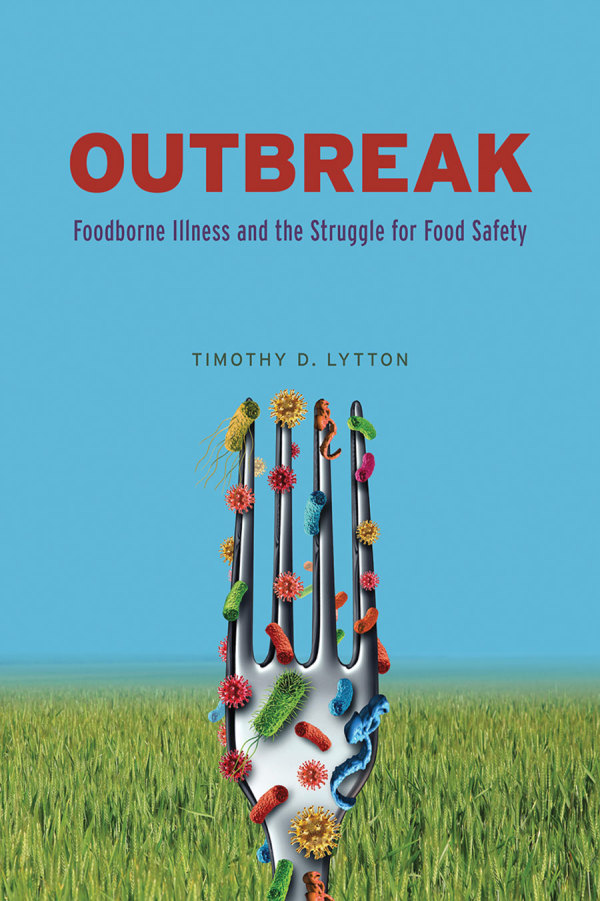
Outbreak: Foodborne Illness and the Struggle for Food Safety

Professor Timothy D. Lytton, a keen scholar of regulatory evolution, provides a lively and well-documented guide to 150 years of major advances in food safety regulation and prevention in the United States. He starts with the early efforts to cleanse and regulate the milk supply in the 19th century that ultimately led to near-universal pasteurization. Efforts to make canned food free of botulism in the 1920s led to a new focus on critical control steps in processing, using sufficient time and heat to eliminate the risk, and thus to a new general approach based on process control. Modernizing meat inspection with process control logic in the 1990s and the recent efforts to make fresh produce safer in the 2000s take the reader to the controversies of the present day.

This book fills a critical gap, weaving the history of public health, regulatory agencies, and the food industry together with issues of immediate concern today. It is an innovative perspective that captures the complexity of the system beyond the scientific report or published regulation. The book should be of interest to students and practitioners of public health and food science and anyone interested in making food reliably safe. 

With fresh examples and detailed interviews, Lytton illustrates the dynamic interplay of outbreak investigations, better prevention strategies developed by industry, consumer advocacy, and regulations. He explains why the resulting balance is a punctuated equilibrium, with longer steady states ending in momentous rapid change. Large and catastrophic outbreaks come as the final trigger, as “focusing events” that, with media coverage, increase public attention and create pressure for change. Lytton tells the striking and less well-known story of what happens behind the scenes as food safety champions within the industry push new solutions and voluntary standards forward, show how they could reduce contamination, and gain adherents up and down the food supply chain, thus leading the way for others in industry and regulators to follow. He also deftly outlines the complex roles of third-party auditors, who provide information to one company about the safety practices of its suppliers, and provides a fresh perspective on the growing role that liability insurers may play in the future.

This is history that uplifts, showing how we honor those who suffered from and died of a foodborne disease that is now preventable in the form of better practices and safer food today. In the crucible of public action, it reminds us all how these advances begin and, with feedback and learning, how they can succeed.

